# Screened AAV variants permit efficient transduction access to supporting cells and hair cells

**DOI:** 10.1038/s41421-019-0115-9

**Published:** 2019-10-15

**Authors:** Xinde Hu, Jinghan Wang, Xuan Yao, Qingquan Xiao, Yuanyuan Xue, Shaoran Wang, Linyu Shi, Yilai Shu, Huawei Li, Hui Yang

**Affiliations:** 1 Institute of Neuroscience, State Key Laboratory of Neuroscience, Key Laboratory of Primate Neurobiology, CAS Center for Excellence in Brain Science and Intelligence Technology, Shanghai Research Center for Brain Science and Brain-Inspired Intelligence, Shanghai Institutes for Biological Sciences, Chinese Academy of Sciences, Shanghai, 200031 China; 2 College of Life Sciences, University of Chinese Academy of Sciences, Beijing, 100049 China; 3 ENT Institute and Otorhinolaryngology Department, Affiliated Eye and ENT Hospital, State Key Laboratory of Medical Neurobiology, Fudan University, Shanghai, China; 4 NHC Key Laboratory of Hearing Medicine (Fudan University), Shanghai, 200031 China

**Keywords:** Biological techniques, Molecular biology, Biological techniques, Molecular biology

Dear Editor,

Genetic malady is a significant pathogenesis underlying sensorineural hearing loss. More than half of the individuals with pre-lingual hearing loss are verified with inherited genetic defects, which are becoming a severe public health issue around the world^1^. Despite the impressive progress in identification of genes associated with deafness, no medical treatment is available for genetic hearing loss except for cochlear implantation. Therefore, developing new treatments that target diverse types of genetic hearing loss is a high priority. Inner ear gene therapy is a promising therapeutic approach to develop treatments for genetic hearing loss. The mammalian cochlea contains two types of hair cells (HCs): inner HCs (IHCs) and outer HCs (OHCs), both of which are essential for the detection and processing of auditory signal^2^. These HCs are surrounded by supporting cells (SCs), a heterogeneous group of cells that are important for cochlear homeostasis^3^. Moreover, SCs are closely associated with genetic defects, resulting in the most common hearing impairments^4,5^. However, the inefficiencies of gene introduction into HCs and SCs not only limit the study of the function of inner ear genes, but also hinder gene therapy application for hereditary deafness. A proper vector for efficient gene delivery into targeted HCs and SCs is a prerequisite for accurate gene therapy.

In the past decade, several gene delivery technologies, including nanoparticles, have been developed for gene delivery into the inner ear. Non-viral nanoparticles, most often made from synthetic and cationic lipid or polymer delivery materials, can be used to deliver genome-editing tools in vitro and in vivo. However, the presence of additional extracellular barriers hinders in vivo application. As a result, most of them have limited gene delivery efficiency in vivo^6,7^.

Adeno-associated virus (AAV) has been certified competent to achieve the safe, efficient, and endurable outcomes of gene therapy. By injection through the round window membrane (RWM), several AAV vectors in different serotypes have been shown to exhibit effective IHCs transduction, but difficultly in targeting the OHCs and SCs remains, leading to only partial correction of hearing in mouse models of inherited deafness^8,9^. Thus, researchers have tried to expand the tropism of viral vectors to simultaneously target cells of various subtypes, including IHCs, OHCs, and SCs. Artificial recombinant AAV vectors have been created to improve infectivity and enhance their ability to avoid neutralization by pre-existing antibodies in the circulatory system^10^. Pseudoserotype vectors, AAV-PHP.eB and AAV-DJ, have been shown to outperform various naturally occurring AAVs in different serotypes in their ability to transduce multiple tissues and organs in vitro and in vivo^10–12^.

In order to screen AAV vectors that target HCs and SCs with high efficiencies, we packaged different subtypes of AAVs (AAV-8, AAV-9, AAV-DJ, and AAV-PHP.eB for HCs; AAV-8, AAV-9, AAV-DJ for SCs) that express tdTomato and separately injected them through the RWM of mice at a dose of 1 × 10^10^ virus genome (vg) (Fig. 1a). Three weeks after injection, modiolar cross-sections of the cochleae showed broad distribution of tdTomato expression in all four serotypes of AAV (Supplementary Fig. S1). TdTomato expression in diverse inner ear cell types, including sensory epithelial, spiral ganglion, spiral ligaments, stria vascularis, and spiral limbus, was observed in all four AAV groups (Supplementary Fig. S1). We further immunostained the sections and found that AAV-8 and AAV-9 infected the IHCs but not the OHCs nor SCs, which was consistent with previous studies (Fig. 1b, d; Supplementary Fig. S2). However, we found that PHP.eB transduced both the IHCs and OHCs effectively (Fig. 1e). Surprisingly, we found that tdTomato expression was detected in SCs in AAV-DJ injection group (Fig. 1f; Supplementary Fig. S2).

**Fig. 1:**
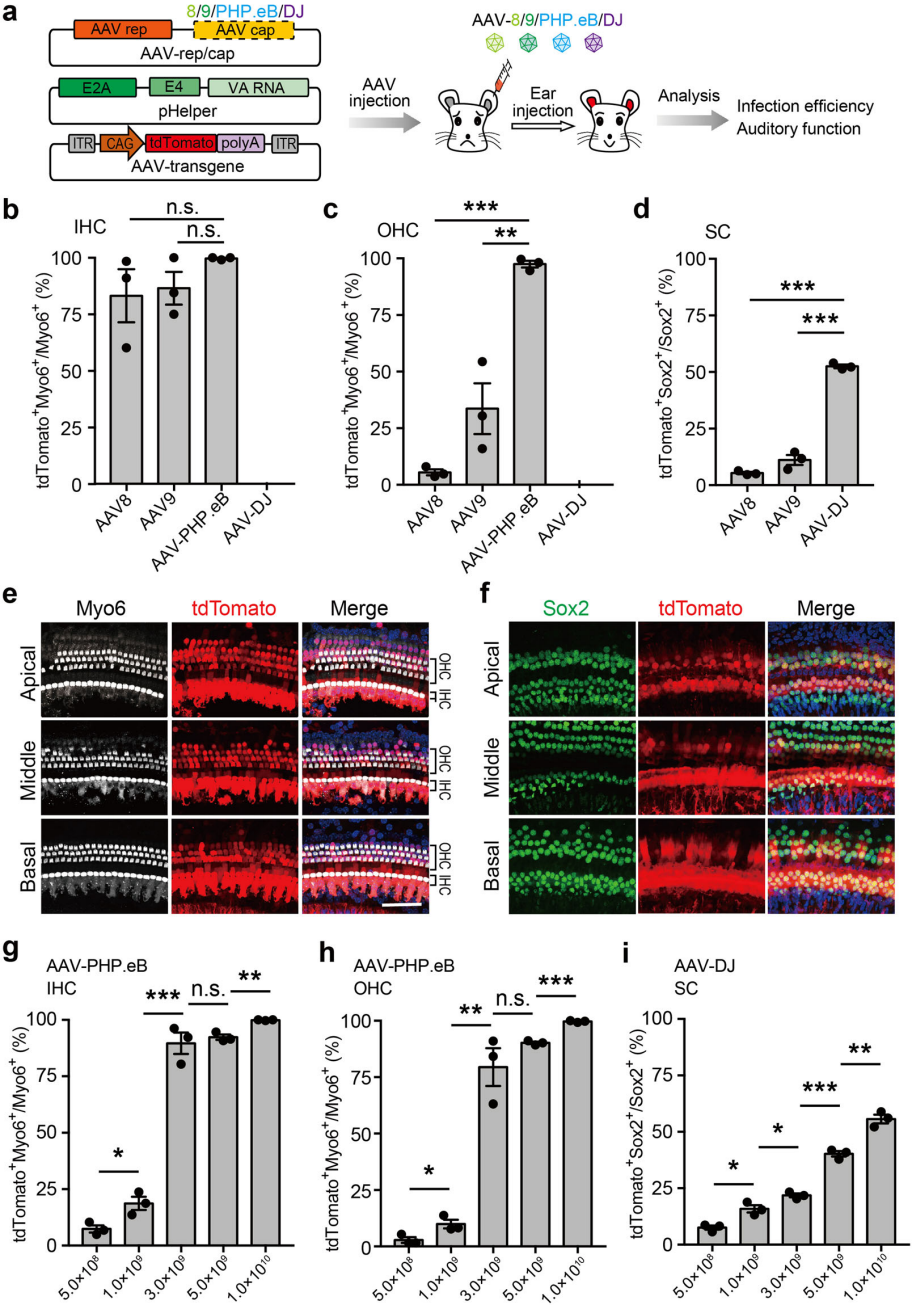
Infection efficiencies of different adeno-associated virus (AAV) variants for hair cells (HCs) and supporting cells (SCs). **a** Schematic overview of infection screening in hair cells and supporting cells using different subtypes of AAVs. Different subtypes of AAVs (AAV-8/9/PHP.eB/DJ) were packaged and injected into the cochlea of P1 Institute of Cancer Research (ICR) mice. The injected cochlea regions were dissected for immunostaining and phenotype analysis at 3 weeks post injection. **b**–**d** Infection efficiencies of different AAV subtypes measured by the percentage of tdTomato^+^ cells in inner HCs (IHCs) (**b**), outer HCs (OHCs) (**c**), and SCs (**d**). Results were obtained from three animals and are presented as mean ± SEM. For each animal, HC and SC infection was quantified at six different locations along the cochlea: two at the apex, two at the middle turn, and two at the cochlear base. *P < 0.05, **P < 0.01, and ***P < 0.001, unpaired Student’s *t* test. **e**, **f** Representative immunofluorescence images of HCs and SCs in cochlea sections injected with AAV-PHP.eB or AAV- DJ at 3 weeks post injection. Myo6, HC marker; Sox2, SC marker; tdTomato, transfected cells. Scale bar, 50 μm. **g**–**i** Infection efficiencies of different doses of AAV-PHP.eB and AAV-DJ in IHCs (**g**), OHCs (**h**) and SCs (**i**). Results were obtained from three animals and are presented as mean ± SEM. *P < 0.05, **P < 0.01, and ***P < 0.001; n.s., no significance; unpaired Student’s *t* test

To evaluate the infection rate of each AAV serotype in different cell types, we conducted immunofluorescence staining on whole-mounted cochleas 3 weeks after injection. We found that AAV-8, AAV-9, and AAV-PHP.eB robustly infected the IHCs, while AAV-DJ showed very rare infection (Fig. 1b, e; Supplementary Fig. S3 and Supplementary Table S1). In the apical turn, tdTomato expression was observed in 98.94 ± 1.30%, 98.41 ± 1.94%, and 100.00 ± 0.00% IHCs in the AAV-8, AAV-9, and AAV-PHP.eB groups, respectively. In the middle and basal turn, AAV-PHP.eB also achieved very high infection efficiency (99.07 ± 1.13% and 100.00 ± 0.00%) (Fig. 1b, e), while AAV-8 and AAV-9 achieved relatively lower infection efficiencies (76.83 ± 27.41 and 73.91 ± 17.15% for AAV-8; 92.05 ± 5.06 and 69.16 ± 20.17% for AAV-9) (Supplementary Table S1).

It is widely accepted that the OHCs are much more difficult to infect with AAV. The infection efficiencies of OHCs using AAV-8 and AAV-DJ were minimal (<5%), and AAV-9 showed a moderate infection efficiency (33.62 ± 13.72%) (Fig. 1c; Supplementary Table S1). By contrast, we found AAV-PHP.eB infected OHCs with a high efficiency, with the average percentages of tdTomato-positive OHCs in the apical, middle, and basal turns being 98.6%, 96.2%, and 97.6%, respectively (Supplementary Table S1). Surprisingly, we found that AAV-DJ showed much higher efficiency (52.51 ± 0.96%) for SC infection than AAV-8 or AAV-9 (5.38 ± 0.63 and 11.10 ± 2.70%) (Fig. 1d, f; Supplementary Fig. S4 and Supplementary Table S1).

To further determine the infection ability of these two AAV serotypes for HCs and SCs, we injected different doses of AAV-PHP.eB and AAV-DJ (5 × 10^8^ to 1 × 10^10^ vg) into the neonatal mouse cochlea. We found that both IHCs and OHCs were infected efficiently with only 3 × 10^9^ vg AAV- PHP.eB (Fig. 1g, h; Supplementary Fig. S5 and Supplementary Table S2); the required AAV dosage was much lower than previously reported serotypes^13^. In addition, we found that AAV-DJ infected up to 50% of SCs with a dose of 1 × 10^10^ vg (Fig. 1i; Supplementary Fig. S6 and Supplementary Table S2).

To determine the safety of AAV-DJ and PHP.eB for gene transfer, we assessed whether the delivery of AAVs had any effect on normal auditory function using the auditory brainstem response threshold test. At all the measured frequencies, we did not observe any difference between the AAV-PHP.eB- or AAV-DJ-injected ears and the contralateral control ears (Supplementary Fig. S7). Therefore, AAV-DJ and PHP.eB demonstrated no obvious toxicity and could be potential AAV vectors for inner ear research and gene therapy.

In summary, we found that different recombinant AAVs exhibited distinctive traits in targeting tropism, and two subtypes of AAVs could achieve high efficiencies of infection in HCs and SCs. AAV-PHP.eB showed extremely high transduction efficacy on both OHCs and IHCs, even at incremental diluted scales. Although several recent studies have validated some new AAV vectors in mouse inner ear HC infection, AAV-PHP.eB is the most efficient AAV vector for gene delivery observed to date. Using high titer AAVs, AAV2.7m8, Anc80L65, and PHP.B can infect about 85%, 65%, and 50% of OHCs, respectively, while AAV-PHP.eB infect nearly 100% of the OHCs at a relatively low dose^13,14^. The infection rate difference of OHCs may due to the affinity of different AAV serotypes. We also demonstrated that AAV-DJ had a relatively high infection efficiency in SCs, surpassing what has been reported previously^15^. Overall, these findings suggest that AAV-PHP.eB and AAV-DJ hold great promise for gene delivery into inner ear, for both basic research and gene therapy purposes.

## Supplementary information

Supplemental Information

## Supplementary information

**Supplementary Information** accompanies the paper at (10.1038/s41421-019-0115-9).
