# Supplementary material for: Screened AAV variants permit efficient transduction access to supporting cells and hair cells
Source: Cell Discov. 2019 Oct 15;5:49. doi: 10.1038/s41421-019-0115-9 (PMC6796865; doi:10.1038/s41421-019-0115-9)
Supplement: Supplementary file 1 — Supplemental Information [file 41421_2019_115_MOESM1_ESM.docx]

**Supplementary Information**

**Supplementary Fig. 1.** Transduction of different AAV serotypes into mouse inner ear

**Supplementary Fig. 2.** Transduction of different AAV serotypes into Organ of Corti

**Supplementary Fig. 3.** AAV-PHP.eB efficient infection of IHCs and OHCs

**Supplementary Fig. 4.** AAV8 and AAV9 infection of SCs in apical, middle, and basal turn of cochlea.

**Supplementary Fig. 5.** PHP.eB Infection ability test using gradient diluted AAV

**Supplementary Fig. 6.** AAV-DJ infection of SCs ability test using gradient diluted AAV.

**Supplementary Fig. 7.** ABR measurement after AAV-PHP.eB and AAV- DJ injection.

**Supplementary Table 1.** Infection efficiencies of different AAV subtypes in IHCs, OHCs, and SCs

**Supplementary Table 2.** Infection efficiencies of AAV-PHP.eB and AAV-DJ with a gradient dose in IHCs, OHCs, and SCs

**Materials and Methods**

**Supplementary Fig. 1.**

**
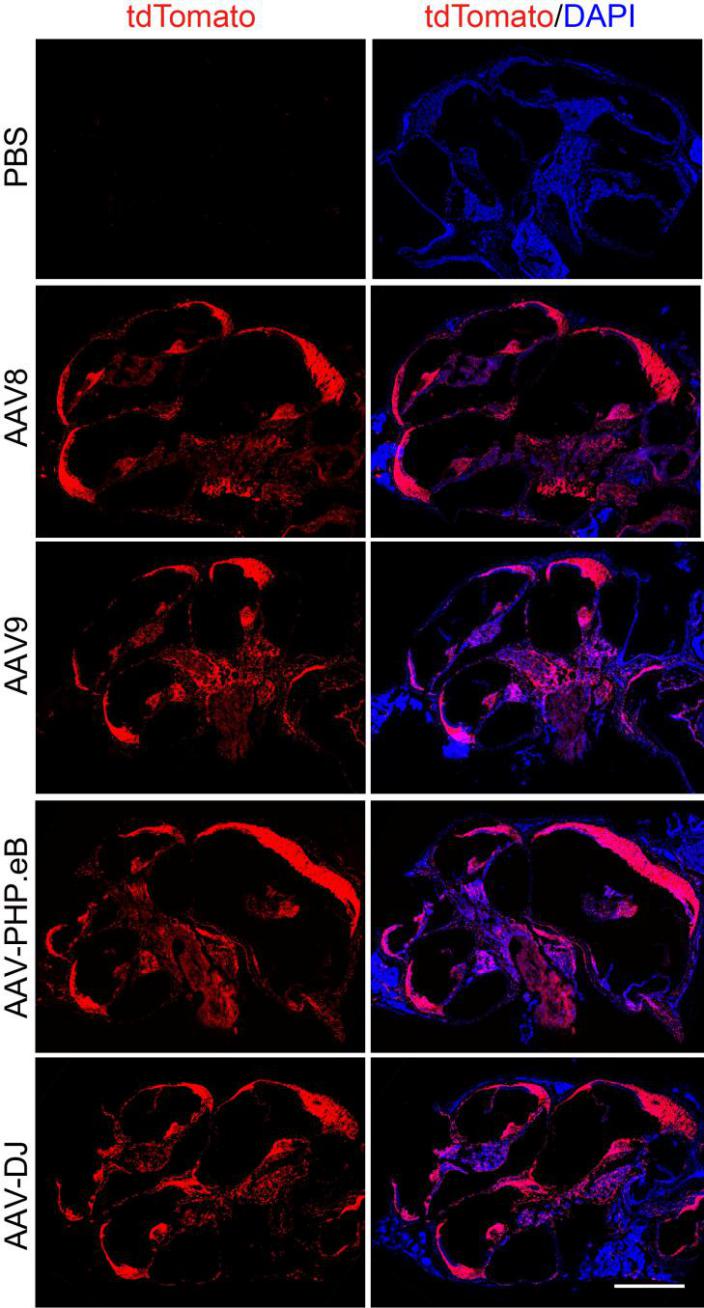
**

**Supplementary Fig. 1.** Transduction of different AAV serotypes into mouse inner ear.

The left ear was injected with the indicated AAVs (AAV8, AAV9, AAV-PHP.eB and AAV-DJ) and the contrallater ear was injected with PBS. tdTomato indicated AAV infection, DAPI shows the gross anatomy structure of inner ear. All four AAVs showed broad infection patterns, AAV8 and AAV9 infected the IHCs, AAV-PHP.eB infected both IHCs and OHCs. Scale bar, 500 μm.

**Supplementary Fig. 2.**

**
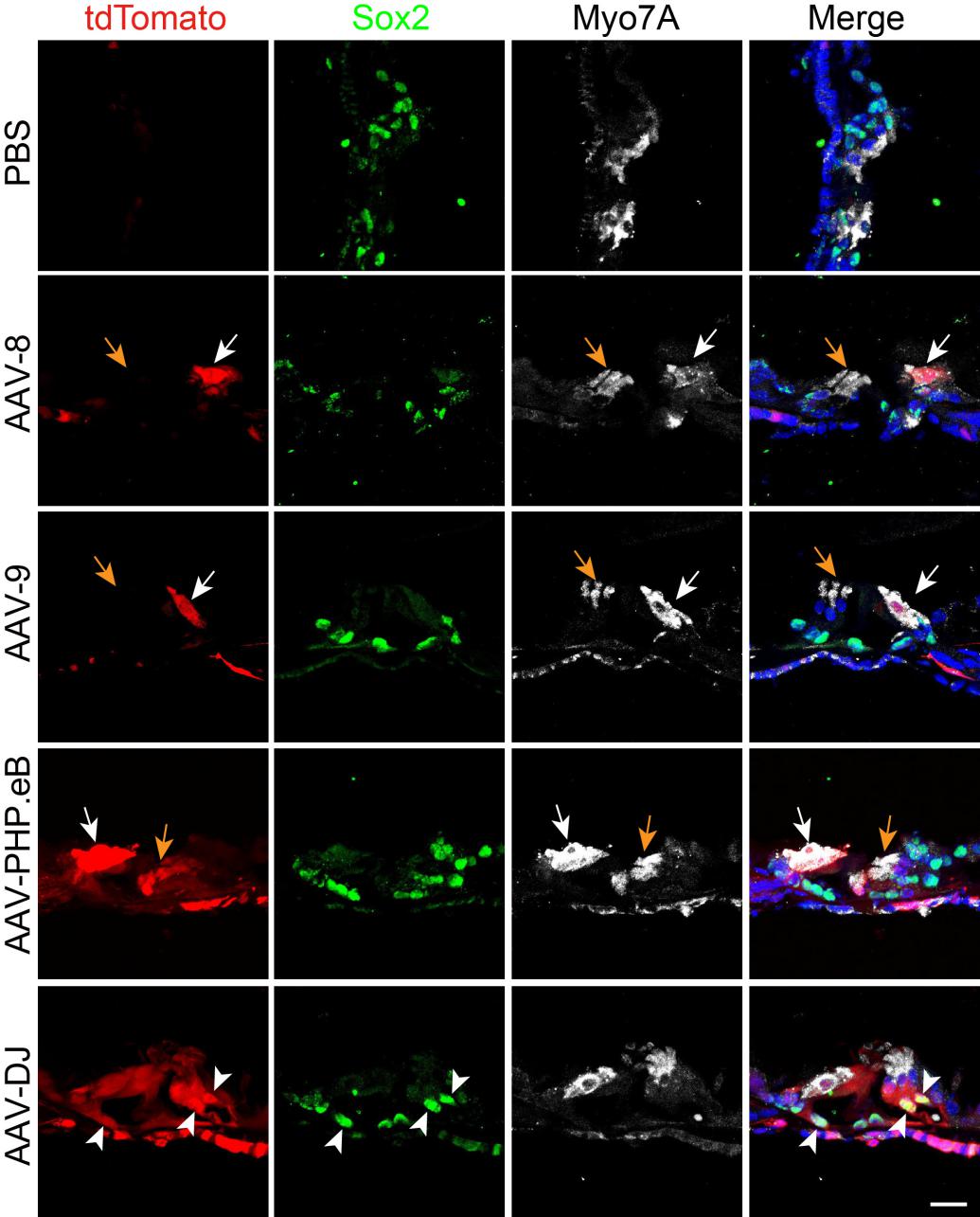
**

**Supplementary Fig. 2.** Transduction of different AAV serotypes into organ of Corti.

The ears injected with AAV8 and AAV9 showed IHC infection, but the OHCs and SCs were not infected. Both IHC and OHC in the AAV-PHP.eB-injection group were infected. AAV-DJ infected the SCs specifically in the organ of corti. The contrallater ear injected with PBS showed no infection of IHC, OHC, or SCs. The white arrows indicate the IHCs, the yellow arrows indicated the OHCs and the white arrowheads indicate the SCs. Scale bar, 20 μm. tdTomato, transfected cells; Sox2, supporting cell marker; Myo7A, hair cell marker. Experiments were independently repeated at least three times per group with similar results.

**Supplementary Fig. 3.**

**
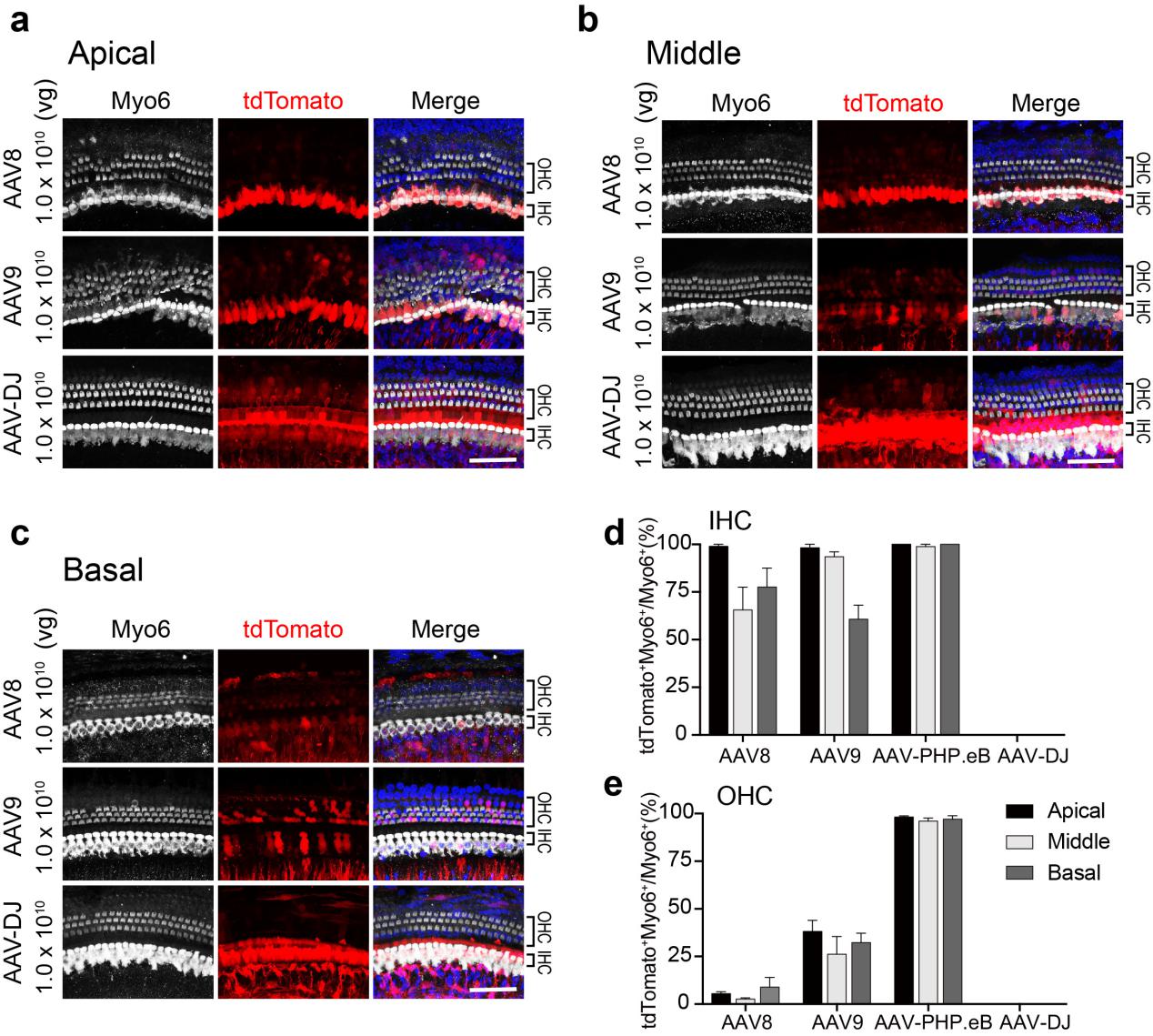
**

**Supplementary Fig. 3.** AAV-PHP.eB efficient infection in IHCs and OHCs.

(a-c) Representative immunofluorescence images of hair cells infection in the cochlea using different subtypes of AAVs. The apical, middle, and basal turn of cochlea were infected with 1.0 × 10^10^ vg of indicated AAV and harvested 3 weeks after injection. Myo6, hair cell marker; tdTomato, transfected cells. Scale bar, 50 μm. (d-e) Infection efficiency of different AAV subtypes were measured by the percentage of tdTomato+ cells in IHCs (d), and OHCs (e). Results were obtained from at least three animals. For each animal, infection of IHCs and OHCs was quantified in at least three different locations.

**Supplementary Fig. 4.**


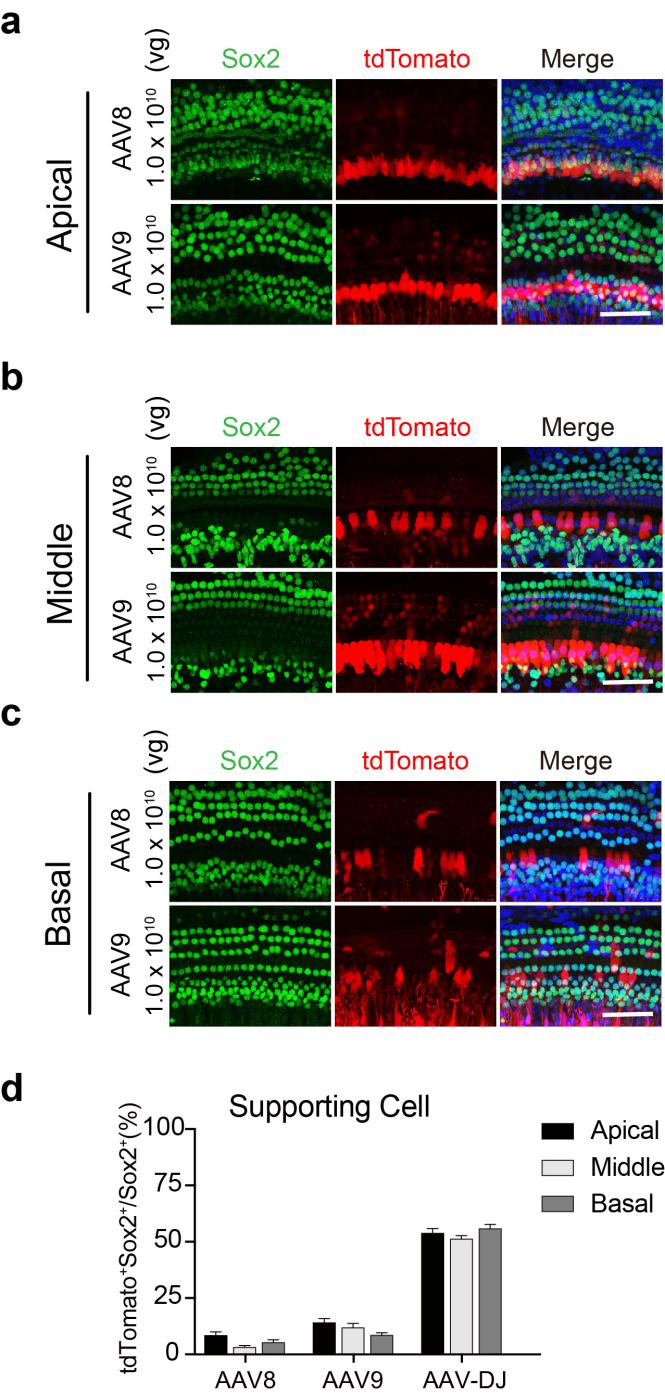


**Supplementary Fig. 4.** AAV8 and AAV9 infection of SCs in apical, middle, and basal turn of cochlea.

(a-c) Representative immunofluorescence images of AAV8 and AAV9 infection of supporting cells in the cochlea**.** Sox2, supporting cell marker; tdTomato, transfected cells. Scale bar, 50 μm. (d) Different AAV subtypes infection efficiency of SCs in apical, middle, and basal turn were measured by the percentage of tdTomato+ cells in Sox2^+^ cells.

**Supplementary Fig. 5.**


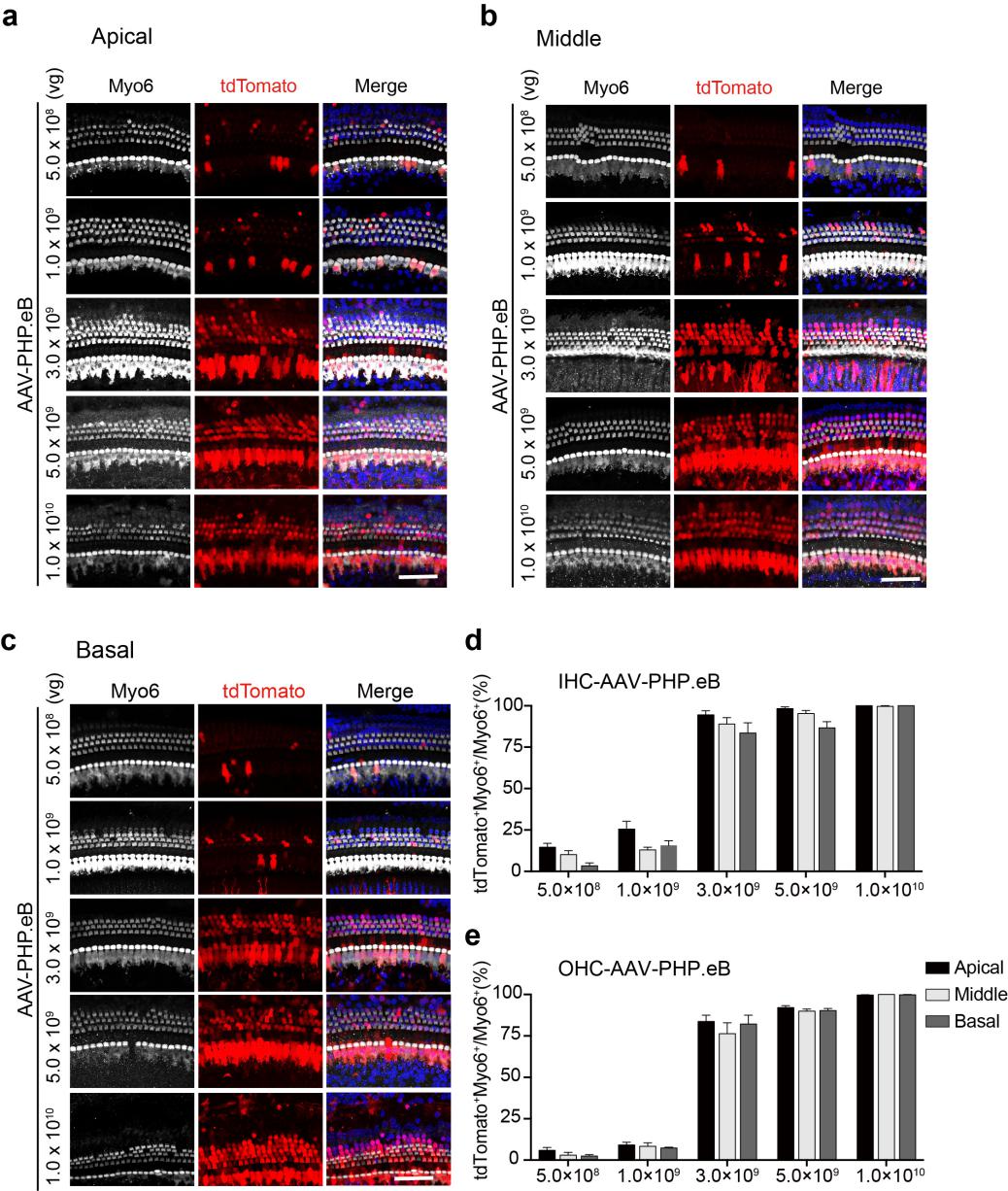


**Supplementary Fig. 5.** PHP.eB Infection ability test using gradient diluted AAV.

(a-c) Representative immunostaining images of hair cells infection in the cochlea using indicated doses of AAV-PHP.eB. Myo6, hair cell marker; tdTomato, transfected cells. Scale bar, 50 μm. (d-e) Infection efficiency of indicated AAV dose was measured by the percentage of tdTomato+ cells in IHCs (d) and OHCs (e). Both IHCs and OHCs were infected efficiently with only 3 x 10^9^ vg AAV- PHP.eB. Results were obtained from at least three animals. For each animal, IHC and OHC infection was quantified in at least three different locations.

**Supplementary Fig. 6.**

**
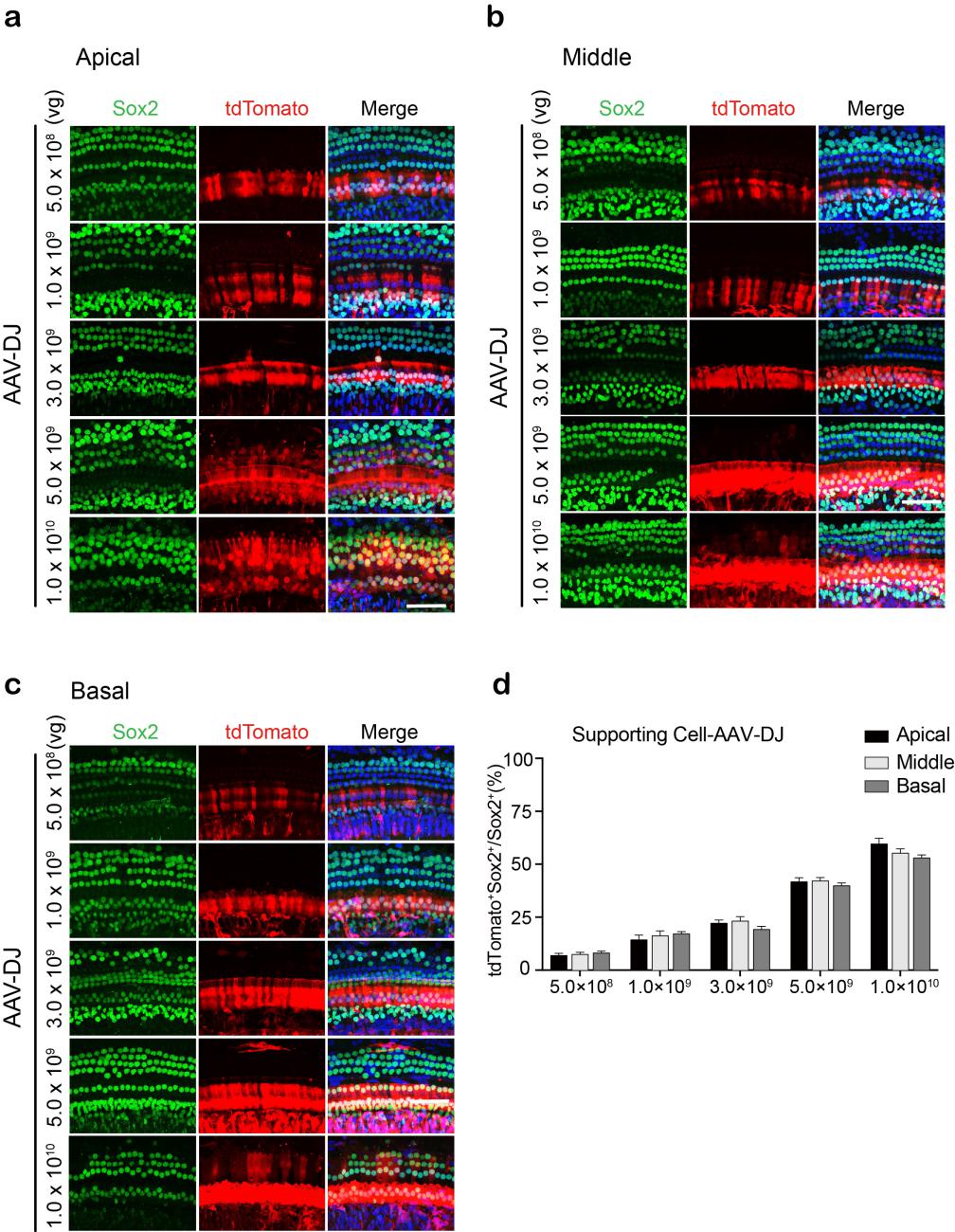
**

**Supplementary Fig. 6.** AAV-DJ infection of SCs ability test using gradient diluted AAV.

(a-c) Representative immunofluorescence images of supporting cells infection in the cochlea using different doses of AAV-DJ. Sox2, supporting cell marker; tdTomato, transfected cells. Scale bar, 50 μm. (d) The infection efficiency of gradient doses of AAV in apical, middle, and basal turn were measured by the percentage of tdTomato+ cells in Sox2+ cells. AAV-DJ showed over 50% infection rate at a dose of 1×10^10^ vg.

**Supplementary Fig. 7.**

**
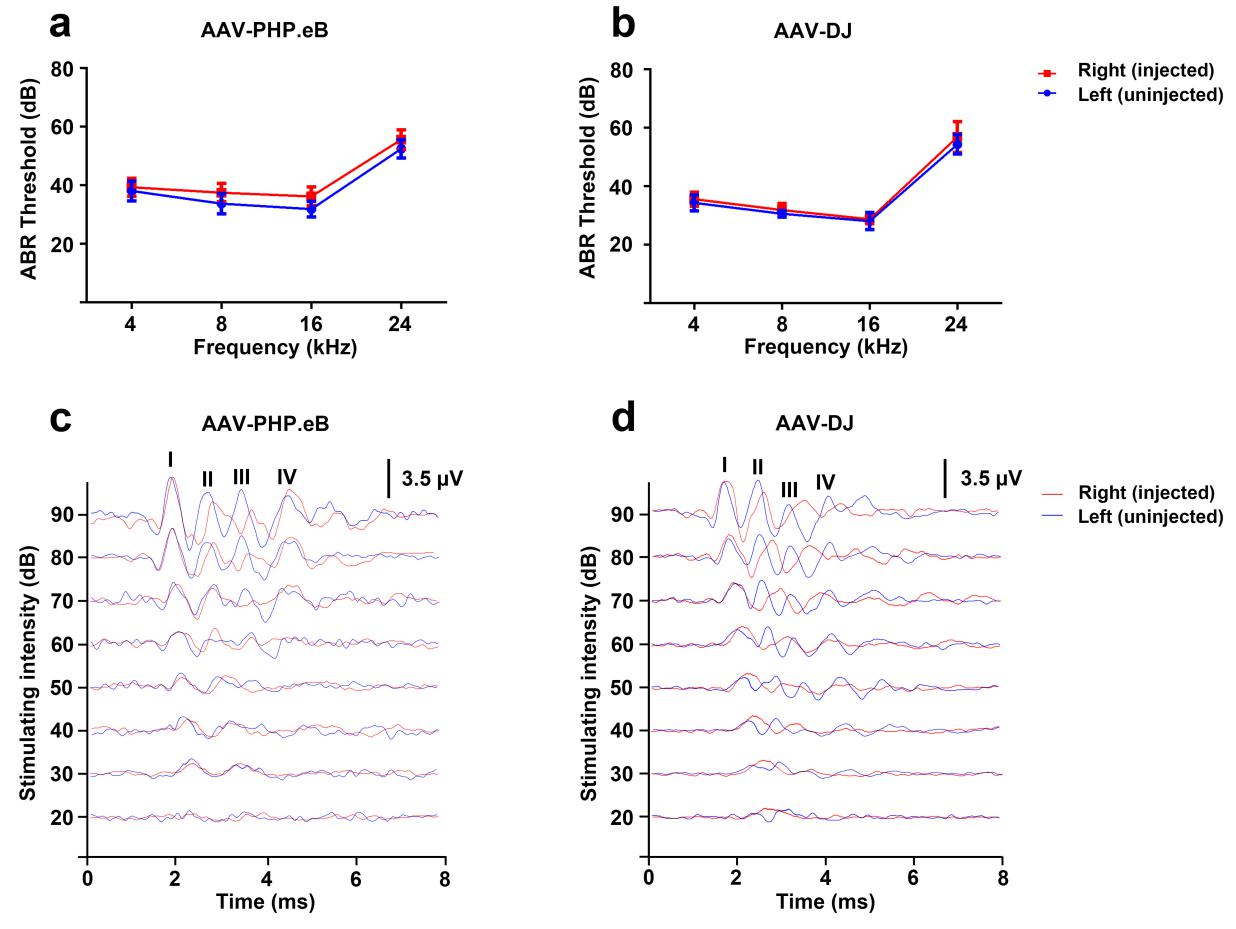
**

**Supplementary Fig. 7.** ABR measurement after AAV-PHP.eB and AAV- DJ injection.

(a-b) ABR thresholds of eight AAV-PHP.eB injected ears (a) and eight AAV-DJ injected ears (b) with the corresponding thresholds of their contralateral uninjected ears. Audiometry measurements were conducted four weeks after AAV injection into the scala tympani of these neonatal subjects. Results are presented as mean±SEM. (c-d) Representative ABR waveforms at 16k Hz in different stimulating intensities of AAV-PHP.eB injected group (c) and AAV-DJ injected group (d), respectively. The peaks of waves I-IV are indicated. The wave I amplitudes of injected and uninjected ears in both AAV-PHP.eB (c) and AAV-DJ (d) injected group are comparable.

**Supplementary Table 1.** Infection efficiencies of different AAV subtypes in IHCs, OHCs and SCs

|  |  | AAV8 | AAV9 | AAV-PHP.eB | AAV-DJ |
| --- | --- | --- | --- | --- | --- |
| IHCs | Apical | 98.94±1.30 | 98.41±1.94 | 100.00±0.00 | 0 |
|  | Middle | 76.83±27.41 | 92.05±5.06 | 99.07±1.13 | 0 |
|  | Basal | 73.91±17.15 | 69.16±20.17 | 100.00±0.00 | 0 |
| OHCs | Apical | 4.47±0.99 | 37.67±12.36 | 98.61±0.91 | 0 |
|  | Middle | 3.07±0.99 | 37.06±21.40 | 96.23±2.29 | 0 |
|  | Basal | 8.94±5.44 | 26.13±11.61 | 97.59±2.95 | 0 |
| SCs | Apical | 8.01±1.51 | 14.57±4.21 | N.D. | 52.44±2.04 |
|  | Middle | 2.66±1.07 | 11.30±2.83 | N.D. | 51.13±1.53 |
|  | Basal | 5.46±3.15 | 7.44±1.13 | N.D. | 53.96±3.44 |

Each subtype of AAV was injected into the cochlea of P1 ICR mice with a dose of 1×10^10^ vg. Results were obtained from at least three animals and are presented as mean ± SEM.

**Supplementary Table 2.** Infection efficiencies of AAV-PHP.eB and AAV-DJ with a gradient dose in IHCs, OHCs, and SCs

|  |  |  | 5.0 x 10^8^ | 1.0 x 10^9^ | 3.0 x 10^9^ | 5.0 x 10^9^ | 1.0 x 10^10^ |
| --- | --- | --- | --- | --- | --- | --- | --- |
| AAV-PHP.eB | IHCs | Apical | 14.00±3.66 | 24.93±11.85 | 95.37±5.67 | 95.99±3.88 | 100.00±0.00 |
|  |  | Middle | 6.49±1.24 | 15.54±3.00 | 91.79±5.22 | 94.91±3.68 | 99.72±0.34 |
|  |  | Basal | 1.64±2.01 | 15.64±3.68 | 81.75±14.58 | 86.06±6.27 | 100.00±0.00 |
|  | OHCs | Apical | 5.61±3.22 | 13.21±9.12 | 81.50±11.14 | 91.56±0.70 | 99.70±0.19 |
|  |  | Middle | 0.35±0.43 | 9.24±2.52 | 75.60±18.70 | 90.12±2.86 | 100.00±0.00 |
|  |  | Basal | 2.51±1.61 | 7.35±0.56 | 81.23±14.13 | 88.99±1.15 | 99.43±0.70 |
| AAV-DJ | SCs | Apical | 6.11±2.45 | 14.29±3.83 | 22.14±2.82 | 39.23±3.15 | 58.54±5.87 |
|  |  | Middle | 7.67±2.46 | 16.19±4.44 | 24.06±3.02 | 41.14±4.09 | 56.75±4.52 |
|  |  | Basal | 8.93±3.78 | 17.18±1.55 | 19.40±3.19 | 40.32±2.77 | 51.83±3.80 |

AAV-PHP.eB and AAV-DJ were each injected into the cochlea of P1 ICR mouse with a gradient dose of 5 x 10^8^, 1 x 10^9^, 3 x 10^9^, 5 x 10^9^ and 1 x 10^10^ vg, respectively. Results were obtained from at least three animals. Data are presented as mean ± SEM.

**Materials and Methods**

**Animal**

P1 ICR mice were used for AAV injection. The use and care of animals complied with the guidelines of the Biomedical Research Ethics Committee of the Shanghai Institutes for Biological Science (CAS).

**AAV production and purification**

Recombinant AAVs were generated by triple transfection of 293T cells (ATCC) using polyethylenimine (PEI). Viral particles were harvested from the media at 72 hrs post transfection and from the cells and media at 120 hrs. Cell pellets were resuspended in 1XGB (10mM Tris with 2mM MgCl_2_, pH=8), freeze-thawed three times, and treated with 100 U/mL Benzonase (Epicentre) at 37 °C for at least 1 hr. Viral media were then adjusted to 500 mM NaCl, incubated at 37 °C for 30 minutes, and clarified by centrifugation at 3,000g. The clarified stocks were then purified over iodixanol (Optiprep, Sigma; D1556) step gradients (15%, 25%, 40%, and 60%). Viruses were concentrated and formulated in phosphate buffered saline (PBS). Virus titers were determined by qPCR with linearized plasmid as a standard. All viruses were stored at -80℃ until use.

**AAV injection**

Mouse pups (P1) were injected via the round window membrane (RWM) using beveled glass microinjection pipettes. Body temperature was maintained on a 38 °C warming pad before surgery. Pups were anesthetized by rapid induction of hypothermia via immersion in ice for 2-3 min until loss of consciousness. The surgical site was disinfected by scrubbing with betadine and wiping with 70% ethanol in three repetitions. A post-auricular incision was made to expose the transparent otic bulla, and a micropipette was advanced manually through the bulla and overlying fascia until the RWM was penetrated by the tip of the micropipette. Approximately 0.6 μL of different subtypes of AAVs (AAV-8/9/PHP.eB/DJ) at the available concentration was injected unilaterally into the ear. After the injection, the skin incision was closed using a black monofilament suture. Pups were subsequently returned to the 38 °C warming pad for 5-10 min and then put back with their mother for continued nursing.

**Immunohistochemistry**

Mice were transcardially perfused with 0.9% saline followed by 4% paraformaldehyde and fixed overnight at 4 °C. The cochleae were then decalcified in 120 mM EDTA for at least 48 h and dissected in pieces for whole-mount immunofluorescence. Samples were rinsed three times in 0.1M phosphate buffer (PB) and incubated with primary antibodies with 5% NGS overnight at 4 °C. The following day, samples were washed three times in PB and then incubated with the secondary antibodies for 2 h at room temperature on an orbital shaker. Finally, the samples were counterstained with DAPI for 20 min and mounted using SlowFade Diamond Antifade Mountant (Thermo Fisher Scientific, S36967) on glass slides. Primary antibodies were goat-anti-Sox2 (Santa Cruz Biotechnology, sc-17320) and rabbit anti-Myosin-VI polyclonal (Proteus Biosciences, 25-6791); secondary antibodies were Alexa Fluor^®^ 488 AffiniPure Donkey Anti-Goat IgG (H+L) (Jackson ImmunoResearch, 705-545-003) and Cy^™^5 AffiniPure Donkey Anti-Rabbit IgG (H+L) (Jackson Immuno Research, 711-175-152).

**Auditory brainstem response (ABR) measurements**

Auditory brainstem responses (ABR) were measured in AAV-PHP.eB and AAV-DJ injected mice at 4 weeks post injection. Bilateral sides of animals were tested, with the uninjected ears as controls (left sides of AAV-PHP.eB and AAV-DJ subjects). Briefly, mice of either sex were anesthetized via intraperitoneal injection of a mixture containing xylazine (10 mg/kg) and ketamine (100 mg/kg), and then three needle electrodes were inserted into the vertex, pinna, and the nasal tip (as the ground). ABR potentials were evoked by 5 ms tone pips (delivered at 35/s), and subsequently were amplified (10,000x), filtered (100 Hz to 3 kHz) and averaged (1,024 responses). The sound stimuli, ranging from 95 dB to 20 dB sound pressure level (SPL), were reduced in 5 dB steps from the maximal level down to 10 dB below threshold. The “threshold” of a certain frequency was defined as the lowest SPL level at which any waveforms could be detected upon visual inspection.

**Quantification statistical analysis**

For the quantification of cochlear supporting cell and hair cell infection efficiency, we randomly captured 2-3 images (1,024 × 1,024 pixel) in each turn (apical, middle and basal turn) using a 60x oil objective (Nikon TiE-A1 plus confocal microscope). The number of supporting cells and hair cells with tdTomato expression were counted in a 800×600 pixel view of each image. The infection rate of each image was measured by the percentage of AAV infected cells (tdTomato^+^) among hair cells (Myo6^+^) or supporting cells (Sox2^+^). The overall infection rate was calculated by averaging the infection rates obtained from the entire cochlea of each mouse. All statistical values are presented as mean ± SEM. Unpaired student’s t test was used to assess the difference of infection efficiency of each AAV group. Differences between data sets were judged to be significant at *P* < 0.05. NIS-Elements Viewer 4.0, ImageJ, Adobe photoshop CS6, and Adobe Illustrator CS6 were used for data analysis and presentation.
